# Tissue doppler echocardiography detects preclinical markers of cardiac lesion in MDS patients

**DOI:** 10.1186/1756-8722-5-30

**Published:** 2012-06-18

**Authors:** Cláudio César Monteiro de Castro, Carlos Bellini Gondim Gomes, Manoel Ricardo Alves Martins, Juliana Cordeiro de Sousa, Silvia MM Magalhaes, Ronald F Pinheiro

**Affiliations:** 1 Post-Graduate Program in Medical Sciences, Federal University of Ceará, Fortaleza, Ceará, Brazil; 2 Post-Graduate Program of Pathology- Federal University of Ceará, Fortaleza, Brazil; 3 R. Pereira Valente, 738, Meireles, 60160250, Fortaleza-Ceará, Brazil

**Keywords:** Myelodysplastic syndrome, Comorbidity, Cardiac dysfunction

## Abstract

Myelodysplastic syndrome (MDS) is a clonal hematopoietic stem cell disorder of elderly people. Cardiac dysfunction is a marker of grim prognosis in MDS. We evaluated cardiac dysfunction of MDS patients with or without transfusion dependency by tissue doppler echocardiography. We found the average values of ventricular end-systolic and end-diastolic volumes in transfusion dependency MDS group higher than others. These results were strongly correlated to hemoglobin levels. Tissue Doppler Echocardiography should be routinely performed in MDS patients to detect preclinical cardiac alterations and prevent more heart insults in this group of chronic anemic aged patients.

## To the Editor

Myelodysplastic syndrome (MDS) is a clonal hematopoietic stem cell disorder and anemia with transfusion dependency is detected in up to 60% of patients [1]. Early recognition of patients at risk of heart failure is difficult because global ventricular function and exercise capacity in chronically transfused patients may remain normal until late in the disease [2].

We evaluated three groups of MDS patients: cases with transfusion dependency (T-MDS), patients without transfusion dependency (NT-MDS) and age-matched controls. Transfusion dependency was considered as reported by Malcovati et al. [3]. Echo-Doppler, tissue velocity imaging and strain measures were obtained using General Electric-Healthcare (GE, Vivid-7) system with a matrix probe M3S.

Parametric data were analyzed by “one-way” analyzes of variance (ANOVA) with Bonferroni’s Multiple Comparison as a post-test. Non-parametric data were analyzed by Kruskal-Wallis. The studies of correlation was assessed by Pearson’s correlation coefficient (r).

**Figure 1:**
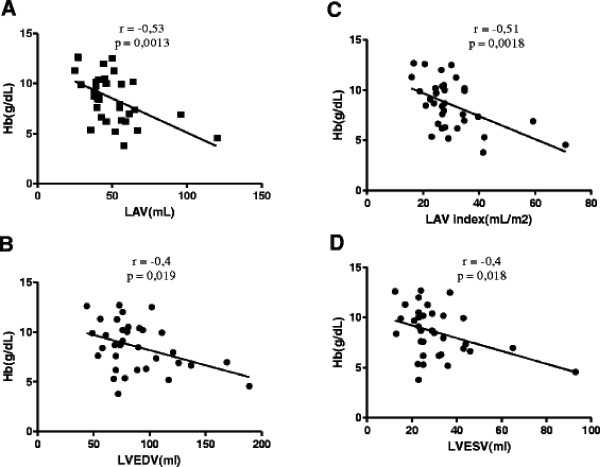
** Linear correlation between left cardiac volumes and values of hemoglobin. A.***LAV: left atrial volume.***B.***LVEDV: left ventricular end-diastolic volume;***C.***LAV index : left atrial volume index.***D.***LVESV: left ventricular end-systolic volume.*

The three groups were composed of 13 T-MDS, 21 NT-MDS and 14 controls. There were no significant differences between groups. See Table 1. Table 2 presents the echocardiographic parameters. The average values of ventricular end-systolic and end-diastolic volumes in T-MDS group were significantly higher than NT-MDS and controls (p <0.05 and p <0.04 respectively). The left atrial volume indexed (LAV index) was significantly larger in patients of T-MDS group than NT-MDS and controls (35.9 ± 15 mL/m^2^, 26.6 ± 5,2 mL/m^2^, 22.8 ± 8 mL/m^2^ respectively) (p <0.004.). A strong correlation between hemoglobin levels and LVEDV (left ventricular end-diastolic volume*)*, LVESV (left ventricular end-systolic volume*)*, LAV (left atrial volume) and LAV index was observed, with **r** values of −0.4, -0.4, -0.53 and 0.51 respectively (p <0.02, p <0.02, p <0.002 and p <0.002 respectively). See Figure 1**.** Otherwise, we found no correlation between ferritin levels and echocardiographic parameters.

**Table 1:** Patients were diagnosed and classified according to WHO, IPSS and WPSS criteria

**Patient**	**WHO**	**IPSS**	**Serum Ferritin**	**Transfusion therapy**	**Transfusional dependent**	**WPSS**
**1**	RA	NA	288,8	No transfusion therapy	NO	NA
**2**	RAEB 2	INT 1	94,7	No transfusion therapy	NO	HIGH
**3**	RCMD	INT 1	416,5	14 RCC	NO	LOW
**4**	RARS	INT 1	1.994,0	69 RCC	Yes	LOW
**5**	RA	NA	826,0	No transfusion therapy	NO	NA
**6**	RA	LOW	22,4	No transfusion therapy	NO	LOW
**7**	MDS-T	NA	3.484,0	42 RCC	Yes	NA
**8**	RARS	NA	1.399,0	64 RCC	Yes	NA
**9**	RCMD	NA	7.107,0	81 RCC	Yes	NA
**10**	RARS	LOW	1.587,0	20 RCC	Yes	LOW
**11**	RARS	INT 1	132,0	04 RCC	NO	INT
**12**	RARS	LOW	1.937,8	82 RCC	Yes	LOW
**13**	RARS	NA	541,0	No transfusion therapy	NO	NA
**14**	RA	LOW	307,0	No transfusion therapy	NO	VERY LOW
**15**	RCMD	INT 1	5.113,1	96 RCC	Yes	INT
**16**	RCMD	LOW	38,0	No transfusion therapy	NO	LOW
**17**	RCMD	NA	98,0	03 RCC	NO	NA
**18**	RARS	LOW	318,0	No transfusion therapy	NO	VERY LOW
**19**	RCMD	LOW	87,0	No transfusion therapy	NO	LOW
**20**	RCMD	NA	765,0	38 RCC	Yes	NA
**21**	RCMD	INT 1	780,0	17 RCC	Yes	HIGH
**22**	RARS	LOW	356,2	No transfusion therapy	NO	VERY LOW
**23**	RCMD	INT 1	298,4	No transfusion therapy	NO	LOW
**24**	RCMD	INT 1	2.160,0	24 RCC	Yes	HIGH
**25**	RA	LOW	86,4	No transfusion therapy	NO	VERY LOW
**26**	RCMD	NA	276,0	No transfusion therapy	NO	NA
**27**	RCMD	NA	1.922,3	24 RCC	Yes	NA
**28**	RAEB 2	INT 2	1.022,0	42 RCC	Yes	VERY HIGH
**29**	RARS	NA	321,0	No transfusion therapy	NO	NA
**30**	RCMD	NA	223,0	12 RCC	Yes	NA
**31**	RAEB 1	INT 1	229,0	03 RCC	NO	VERY LOW
**32**	RCMD	INT 1	132,4	No transfusion therapy	NO	INT
**33**	RCMD	NA	556,0	04 RCC	NO	NA
**34**	RCMD	NA	850,0	10 RCC	NO	NA

Legend. MDS unclassifiable; RA, refractory anemia; RAEB, RA with excess of blasts; RARS, RA with ringed sideroblasts; RCMD, refractory cytopenia with multilineage dysplasia; t-MDS, therapy-related MDS; WHO, World Health Organization. red cell concentrate – RCC. NA -Not applicable, due to cytogenetics by G-banding without metaphases).

**Table 2:** Echocardiographic parameters of patients and controls

	***Controls(14)***	***NT-MDS(21)***	***T-MDS(13)***	***P value***
**Baseline demographics and characteristics**				
*Age (year)*	72.4 ± 8 (58–84)	70.3 ± 14 (47–88)	65.2 ± 20 (27–90)	NS
*Gender (m/f)*	(6/8)	(7/14)	(6/7)	
*Body weigth (kg)*	66.0 ± 11.9 (46 – 83)	63.7 ± 10.1 (43.6 – 91.3)	64.9 ± 10.1 (49.3 – 90)	NS
*Height (m)*	1.59 ± 0.09 (1.45 – 1.75)	1.57 ± 0.07 (1.46 – 1.72)	1.59 ± 0.08 (1.45 – 1.70)	NS
*BSA (m*^*2*^*)*	1.68 ± 0.16 (1.42 – 1.98)	1.64 ± 0.15 (1.33 – 2.01)	1.67 ± 0.15 (1.4 – 2.01)	NS
*BMI (kg/m*^*2*^*)*	26.2 ± 5.7 (19.1 – 36.9)	25.8 ± 2.9 (20.5 – 32.3)	25.5 ± 3.7 (21.4 – 32.7)	NS
*HR (bpm)*	75.8 ± 5.4 (66–83)	77.6 ± 1.4 (66–88)	78.4 ± 2.2(65–90)	NS
*SBP (mmHg)*		139.2 ± 19 (110–180)	121.8 ± 16 (100–150)	<0.02
*DBP (mmHg)*		77.9 ± 12 (60–108)	70 ± 9.6 (60–90)	NS
*Hb (g/dL)*		9.85 ± 1.8 (6.6 - 12.7)	6.5 ± 1.7 (3.8 - 9.9)	<0.001
*Ferritin (ng/mL)*		298.8 ± 234 (22.4 a 850)	2269 ± 1931 (223 a 7101)	<0.001
***Chamber quantification and ejection fraction of patients and controls***				
*LVDD (mm)*	*46.0 ± 4.7 (40–57)*	*48.5 ± 3.9 (41–56)*	*49.3 ± 6.6 (40–64)*	*NS*
*LVSD (mm)*	*26.9 ± 4.9 (19–39)*	*27.7 ± 3.7 (22–37)*	*29.5 ± 6.8 (23–50)*	*NS*
*IVS (mm)*	*8.9 ± 2.3 (6–16)*	*8.5 ± 0.9 (7–11)*	*8.9 ± 1.4 (7–12)*	*NS*
*LVPW (mm)*	*8.5 ± 1.7 (6–13)*	*8.4 ± 0.7 (7–10)*	*8.8 ± 1.4 (7–12)*	*NS*
*MASS (g)*	*165.9 ± 61,0 (79–326)*	*173.8 ± 34.1 (116–249)*	*165.9 ± 61.0 (90–321)*	*NS*
*MASS index (g/m*^*2*^*)*	*101.3 ± 37 (55.6 - 185)*	*106 ± 18 (73–140.7)*	*110 ± 37.1 (56.2 - 198)*	*NS*
*EF%TEI*	*71.8 ± 7.6 (58.2 - 89)*	*71.9 ± 6.5 (58.8 - 80.1)*	*69.8 ± 9.3 (43.6 - 83.9)*	*NS*
*FS (%)*	*41.4 ± 6.8 (31.2 - 58)*	*41.6 ± 5.7 (31–48.7)*	*39.4 ± 7.2 (22–53)*	*NS*
*LA (mm)*	*32 ± 4.2 (27–44)*	*33.4 ± 4.2 (28–43)*	*36.5 ± 4.9 (31–46)*	<0.04*
*EF%SIM*	*67.7 ± 7.3 (50.1 - 81.3)*	*66.2 ± 4.8 (55.2 - 76.1)*	*64.7 ± 5.4 (50.7 - 70.8)*	*NS*
*LVEDV (ml)*	*65.5 ± 18 (37–105)*	*85.1 ± 29.9 (44–169)*	*92.8 ± 36.1 (49–189)*	<0.05*
*LVESV (ml)*	*20.5 ± 6.2 (10–28)*	*28.7 ± 11.9 (12.5 - 65)*	*33.8 ± 19.7 (15–93)*	<0.04*
*LAV (ml)*	*39.1 ± 14.5 (17–69)*	*43.5 ± 10.3 (25–64)*	*59.8 ± 24.8 (29–120)*	<0.006**
*LAV index (mL/m*^*2*^*)*	*22.8 ± 8 (10.5 - 39.6)*	*26.5 ± 5.2 (15.9 - 34.8)*	*35.9 ± 15 (18.8 - 70.9)*	<0.004**
***Doppler parameters (transmitral and myocardial tissue) and strain of patients and controls***				
*Evel(cm/s)*	77.2 ± 13.2 (55.4 - 105)	89.6 ± 20 (63–128)	96.6 ± 14.2 (68.5 - 116)	<0.02*
*Avel(cm/s)*	94.3 ± 18 (68.6 - 137.3)	100.2 ± 19 (68.5 - 131)	100.6 ± 27 (52.8 - 145)	NS
*E/A*	0.83 ± 0.2 (0.6 - 1.2)	0.9 ± 0.2 (0.6 - 1.4)	1.0 ± 0.26 (0.64 - 1,6)	NS
*Em(cm/s)*	8.6 ± 3.2 (4.1 - 14)	9.3 ± 2.4 (5.4 - 14)	10.4 ± 2.5 (7.5 a 14.8)	NS
*E/Em*	10 ± 3.4 (5.3 - 16.2)	10.3 ± 3,9 (5.6 - 20)	9.7 ± 2.9 (5.9 - 14.4)	NS
*LV-Sm(cm/s)*	6.5 ± 1.3 (5.1 - 9.5)	7.9 ± 1.3 (5.5 - 10.8)	7.8 ± 1.3 (6–10,3)	<0.02*
*RV-Sm(cm/s)*	10.1 ± 0.7 (9.1 - 11)	11.4 ± 3.3 (7.6 - 19)	12.3 ± 1,5 (9.8 - 14,7)	NS
*TAPSE(mm)*	24.9 ± 4.2 (20–33)	28.2 ± 5.3 (21–39)	28.9 ± 5 (22–40)	NS
*VD basal(mm)*	30.1 ± 6.4 (22–48)	31.7 ± 4 (24–41)	31.5 ± 4 (23–39)	NS
*ST2DL (%)*	−19.9 ± 2.7 (−24 to −13)	−20.7 ± 2.5 (−25 to −16,8)	−20.9 ± 1.4 (−23 to - 18,6)	NS

Legend. NT-MDS: non-transfused patients; T-MDS: transfused patients; LVDD: left ventricular diastolic diameter; LVSD: left ventricular systolic diameter; IVS: inter-ventricular septum; LVPW: left ventricular posterior wall; MASS: left ventricular mass; EF%TEI: ejection fraction Teicholz; FS: fractional shortening; LA: left atrial diameter; EF%SIM: ejection fraction Simpson; LVEDV: left ventricular end-diastolic volume; LVESV: left ventricular end-systolic volume; LAV: left atrial volume.

The reduction of blood viscosity in severe anemia increases blood return [4] and ventricular preload which lead to atrial and ventricular enlargement observed in T-MDS patients. Confirming this hypothesis, these results are correlated to hemoglobin levels.

The T-MDS group showed no clinical sign of cardiac dysfunction. Otherwise, cardiac alterations were detected by tissue-doppler echocardiography, a relative fast and cheap bedside method to evaluate heart function. Echocardiography should be routinely performed in MDS patients to detect preclinical cardiac alterations and prevent more heart insults in these group of chronic anemic aged patients.

## Abbreviation

NT-MDS, Non-transfused patients; T-MDS, Transfused patients; LVDD, Left ventricular diastolic diameter; LVSD, Left ventricular systolic diameter; IVS, Inter-ventricular septum; LVPW, Left ventricular posterior wall; LVEDV, Left ventricular end-diastolic volume; LVESV, Left ventricular end-systolic volume; LAV, Left atrial volume; RCC, Red cell concentrate; VD, Ventricular dysfunction.

## Competing interests

The authors declare that they have no competing interests.

## Authors’ contributions

CCMC was the principal investigator and takes primary responsibility for the paper. CBGG provide technical support. MRAM participated in the statistical analysis. JCS performed the laboratory work for this study and edited the manuscript. SMMM provided critical revision. RFP coordinated the research and wrote the paper.
